# Different Region Analysis for Genotyping *Yersinia pestis* Isolates from China

**DOI:** 10.1371/journal.pone.0002166

**Published:** 2008-05-14

**Authors:** Yanjun Li, Erhei Dai, Yujun Cui, Min Li, Yujiang Zhang, Mingshou Wu, Dongsheng Zhou, Zhaobiao Guo, Xiang Dai, Baizhong Cui, Zhizhen Qi, Zuyun Wang, Hu Wang, Xingqi Dong, Zhizhong Song, Junhui Zhai, Yajun Song, Ruifu Yang

**Affiliations:** 1 Laboratory of Analytical Microbiology, State Key Laboratory of Pathogen and Biosecurity, Institute of Microbiology and Epidemiology, Beijing, China; 2 Qinghai Institute for Endemic Diseases Prevention and Control, Xining, China; 3 The Center for Disease Control and Prevention of Xinjiang Uygur Autonomous Region, Xinjiang Uygur Autonomous Region, China; 4 Yunnan Institute for Endemic Disease Control and Prevention, Yunnan, China; Max Planck Institute for Evolutionary Anthropology, Germany

## Abstract

**Background:**

DFR (different region) analysis has been developed for typing *Yesinia pestis* in our previous study, and in this study, we extended this method by using 23 DFRs to investigate 909 Chinese *Y. pestis* strains for validating DFR-based genotyping method and better understanding adaptive microevolution of *Y. pestis*.

**Methodology/Principal Findings:**

On the basis of PCR and Bionumerics data analysis, 909 *Y. pestis* strains were genotyped into 32 genomovars according to their DFR profiles. New terms, Major genomovar and Minor genomovar, were coined for illustrating evolutionary relationship between *Y. pestis* strains from different plague foci and different hosts. *In silico* DFR profiling of the completed or draft genomes shed lights on the evolutionary scenario of *Y. pestis* from *Y. pseudotuberculosis*. Notably, several sequenced *Y. pestis* strains share the same DFR profiles with Chinese strains, providing data for revealing the global plague foci expansion.

**Conclusions/significance:**

Distribution of *Y. pestis* genomovars is plague focus-specific. Microevolution of biovar Orientalis was deduced according to DFR profiles. DFR analysis turns to be an efficient and inexpensive method to portrait the genome plasticity of *Y. pestis* based on horizontal gene transfer (HGT). DFR analysis can also be used as a tool in comparative and evolutionary genomic research for other bacteria with similar genome plasticity.

## Introduction

Plague, one of the most devastating infections in the human history, is a reemerging zoonotic disease that is transmitted to humans from natural rodent reservoirs, commonly via the bite of an infected flea. *Yersinia pestis*, the causative agent of plague, has killed hundreds of millions of people in the three major historical plague pandemics[1]. As a typical biological warfare agent, *Y. pestis* might be used in the war or as a bioterrorism agent in future, which poses significant threats on the health and safety of our human beings[2].

*Y. pestis* has been shown to evolve from *Y. pseudotuberculosis* serotype O1:b within the last 20,000 years [3], [4]. The very short evolutionary history of *Y. pestis* accounts for the limited phenotypic and genetic diversities. *Y. pestis* has been traditionally classified into three biovars according to their ability to reduce nitrate and utilize glycerol: Antiqua (positive for both), Medievalis (negative for nitrate reduction and positive for glycerol utilization), and Orientalis (positive for nitrate reduction and negative for glycerol utilization). Recently, a new biovar Microtus was proposed by whole genome sequencing and genetic analysis[5], [6]. *Y. pestis* is a multi-host and multi-vector pathogen, involving more than 200 species of wild rodents as host and over 80 species of fleas as vector [7]. Different hosts and vectors have their own specific ecological landscape to inhabit, shaping various niches for *Y. pestis*. During its expansion and adaptation to new niches, *Y. pestis* undergoes considerable genetic variations in its genome to balance the natural selection, which can partly explain the genome diversity of the strains from different plague foci. Figuring out the genome diversity of *Y. pestis* will help us better understand the origin and expansion of plague, and provide us solid data for developing reliable genotyping system for this bacterium.

Genotyping is based on genetic variations of target microorganisms. Different methods have been applied to *Y. pestis* for this purpose, such as PFGE(pulsed-field gel electrophoresis)[8], [9], MLST(multilocus sequence typing)[4], VNTR(variable number of tandem repeat)[10], [11], [12], Ribotyping[13], RAPD(randomly amplified polymorphism DNA)[14], [15], IS (insertion sequence) based typing [4], [16], and PCR-based technique as well[17]. DFR typing is an alternative typing method for *Y. pestis*. The term DFR (different region) is coined to describe a genomic region present in some strains and absent in other strains of the same species [18]. By *in silico* comparative genomics and DNA microarray analysis, a set of different regions (DFRs) were identified in the genomes of different *Y. pestis* strains [19], [20]. In our previous study, a genotyping system based on 22 DFRs disclosed 14 genomovars (termed to describe genotypes based on DFR profiles) among 260 Chinese isolates of *Y. pestis* [20].

In this study, the previous described 22 DFRs and DFR23 identified by SSH(suppression subtractive hybridization) [21] were used to investigate more Chinese *Y. pestis* strains for validating DFR-based genotyping system. We also proposed the new terms, Major genomovar and Minor genomovar, to describe the region-specific distribution of DFR profiles.

## Results and Discussion

### DFR profiling of 909 Chinese strains of *Y. pestis*

In this study, we initially included 912 Chinese isolates of *Y. pestis*. As we know, DFR01, DFR02 and DFR03 locate in the plasmid pMT1[20]. Screening these strains with primers specific for this plasmid identified 3 pMT1-negative strains. The negative results of the DFR01-03 in these strains are due to the loss of plasmid pMT1, which is different from the absence on the basis of HGT in the pMT1-positive strains. Therefore, the pMT1-negative strains are not suitable for evaluating this genotyping system, and should be excluded from this study. The pMT1 is a virulence-associated plasmid of *Y. pestis*, and counts for the phenotype of F1 antigen. Although F1^−^ strains have been constructed in laboratory, natural *Y. pestis* isolates with an F^−^ phenotype appear to be exceedingly rare[1]. Detailed analysis of these pMT1^−^ strains might be helpful to understand its introduction into or loss from *Y. pestis*.

The remaining 909 *Y. pestis* strains were then analyzed for the 23 DFRs profiles. Our previous study has grouped 260 strains into 14 genomovars (genomovar01 to genomovar14) by 22 of 23 DFRs[20]. These 260 strains were also included in this study, and tested by the DFR23-specific primers. For those 14 genomovars, we only found DFR23 in genomovar01, 02 and 03. The previously described genomovar01 strains were grouped as two subgroups by the presence or absence of DFR23. To maintain the consistency of this typing system, we named the DFR23^+^ strains as genomovar01a and the DFR23^−^ strains as genomovar01b. All of the genomovar02 and genomovar03 strains harbor DFR23, so we still reserve these names for the corresponding strains. The newly identified genomovar were serially named from genmovar15 to genmovar31.

**Figure 1:**
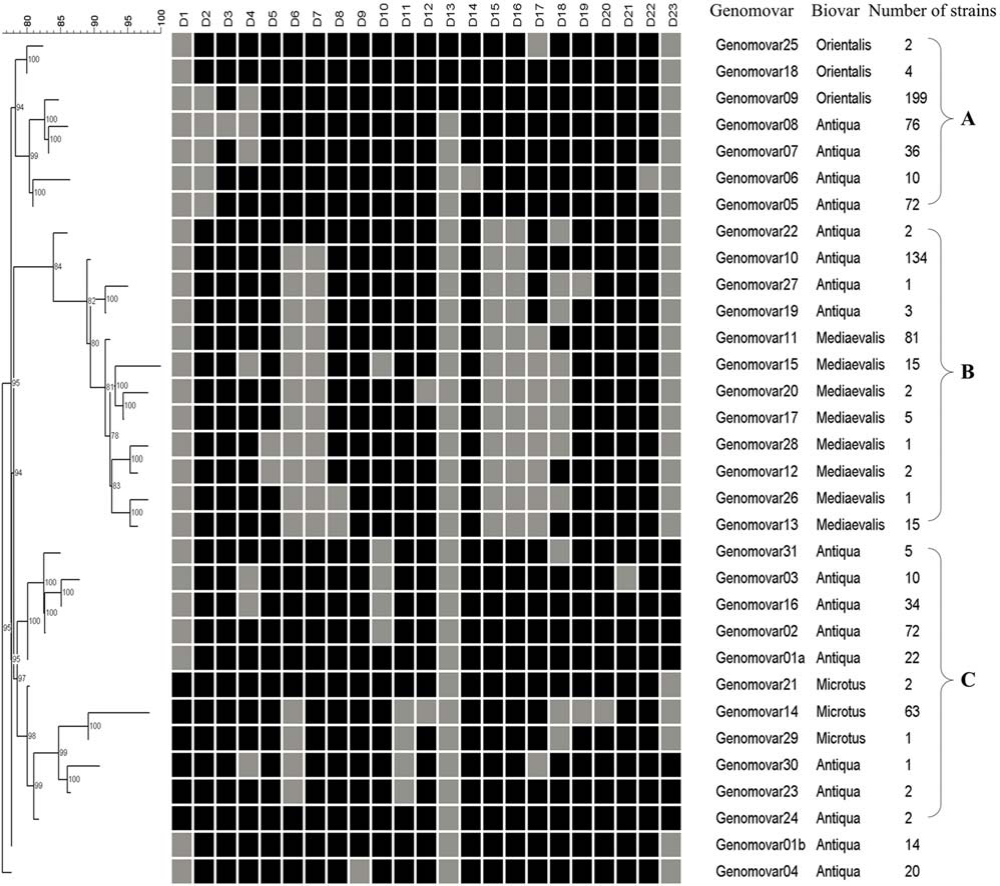
Neighbor-Joining Dendrogram of the 32 genomovars based on DFR profiles. The black and grey squares indicated the presence and absence of the corresponding DFRs, respectively. There are three clusters (A, B and C) for most of the genomovars.

The 909 strains were grouped into 32 genomovars. The DFR profiles and Neighbor-Joining dendrogram of the 32 genomovars were shown in Figure 1. Most of the genomovars were clustered into 3 clusters, namely A, B and C, except for genomovar01b and genomovar04. All Orientalis strains (205 strains in 3 genomovars) were grouped together in cluster A, all Medievalis strains (122 strains in 8 genomovars) in cluster B and Microtus strains (66 strains in 3 genomovars) in cluster C. This clearly illustrated the close relationship of strains of the same biovar in China. However, Antiqua strains (516 strains in 18 genomovars) were distributed in different branches of all 3 clusters, revealing considerable genome diversities of Antiqua strains. This is not the first time to find this fact. SNPs(Single nucleotide polymorphisms) analysis has identified 2 different molecular groups of Antiqua strains on 2 evolutionary lineages of *Y. pestis* (1.ANT and 2.ANT), which fitted into Orientalis and Medievalis branches, respectively [3]. CRIPSR analysis also identified 2 clusters of Antiqua strains (Asian and African)[22]

Biovar system is based on 2 phenotypes (nitrate reduction and glycerol utilization). Several studies have reported that, nitrate reduction negative stains might have different genetic mechanism for this phenotype[3], [6]. As biovar strains are not genetically homogeneous, it seems that biovar typing system is no longer suitable for evolutionary or taxonomic purpose. Some genetics-based systems, such as SNP- and DFR-based ones, are alternatives as reliable typing methods for *Y. pestis* [3], [4], [20].

### Major genomovar and Minor genomovar

In China, there are 15 plague foci covering more than 1.4 million square kilometers now. A large number of *Y. pestis* strains with clear background were isolated from different plague foci since the year of 1943. However, no strain was isolated since 1956 from animals during daily surveillance in *Marmota sibirica* Plague Focus of the Hulun Buir Plateau in Inner Mongolia (Focus N), where used to have plague epidemics in early 19^th^ century. There was also no report of animal plague epidemics since then. We call Focus N a silent focus. The 909 strains in this study were carefully selected, considering their phenotypes, years and locations of isolation, vectors and hosts, etc. We assumed that they could represent the most abundant diversities of Chinese *Y. pestis* strains.

Table 1 showed the distribution of genomovars in different plague foci. In our previous study, 260 strains were genotyped into 14 genomovars. Although we added nearly 650 strains and one new DFR marker in this study, we only got 18 new genomovars. More interestingly, most of our new strains fell into the previously identified genomovars (826/909, 90.9%), and most of the new genomovars contained only a few strains. Fourteen genomovars comprised more than 10 strains, which cover 93.8% (853/909) of all the tested strains. The DFR typing system is still open to new markers and new strains. It seems that, by adding more markers, we can get higher resolution without disturbing the framework of DFR typing.

**Table 1:** Distribution of genomovars in 909 strains of *Y. pestis* isolated from China

Focus or Subfocus	Number of strains	Genomovar*																															
		01a	01b	02	03	04	05	06	07	08	09	10	11	12	13	14	15	16	17	18	19	20	21	22	23	24	25	26	27	28	29	30	31
A	13	0	0	0	0	***11***	0	0	0	0	0	0	0	0	0	0	0	1	0	0	0	0	0	0	0	0	1	0	0	0	0	0	0
B1	14	0	1	03	0	***09***	0	0	0	0	0	0	0	0	0	0	0	0	0	0	0	0	0	0	0	0	0	0	0	0	0	0	1
B2	46	2	0	***43***	0	0	0	0	0	0	0	0	0	0	0	0	0	1	0	0	0	0	0	0	0	0	0	0	0	0	0	0	0
B3	71	3	0	***24***	10	0	0	0	0	0	0	0	0	0	0	0	0	***32***	0	0	0	0	0	0	0	0	0	0	0	0	0	0	2
B4	17	***15***	0	2	0	0	0	0	0	0	0	0	0	0	0	0	0	0	0	0	0	0	0	0	0	0	0	0	0	0	0	0	0
C	152	0	9	0	0	0	***63***	10	10	***48***	0	8	0	0	0	0	0	0	0	0	1	0	0	0	1	2	0	0	0	0	0	0	0
D	33	0	2	0	0	0	3	0	0	***27***	0	0	0	0	0	0	0	0	0	0	0	0	0	0	1	0	0	0	0	0	0	0	0
E	31	0	0	0	0	0	0	0	***23***	0	8	0	0	0	0	0	0	0	0	0	0	0	0	0	0	0	0	0	0	0	0	0	0
F	198	0	0	0	0	0	0	0	1	1	***191***	0	0	0	0	0	0	0	0	3	0	0	0	0	0	0	1	1	0	0	0	0	0
G	44	0	1	0	0	0	0	0	0	0	0	***39***	0	0	0	0	0	0	1	0	1	0	0	2	0	0	0	0	0	0	0	0	0
H	91	0	0	0	0	0	0	0	1	0	0	***87***	0	0	0	0	0	0	1	1	0	0	0	0	0	0	0	0	1	0	0	0	0
I	74	0	0	0	0	0	0	0	0	0	0	0	***69***	2	0	0	0	0	0	0	1	2	0	0	0	0	0	0	0	0	0	0	0
J	17	0	0	0	0	0	0	0	1	0	0	0	0	0	***15***	0	0	0	1	0	0	0	0	0	0	0	0	0	0	0	0	0	0
K1	14	1	0	0	0	0	0	0	0	0	0	0	***12***	0	0	0	0	0	0	0	0	0	0	0	0	0	0	0	0	1	0	0	0
K2	11	1	1	0	0	0	*6*	0	0	0	0	0	0	0	0	0	0	0	1	0	0	0	0	0	0	0	0	0	0	0	0	0	2
L	22	0	0	0	0	0	0	0	0	0	0	0	0	0	0	***20***	0	0	1	0	0	0	0	0	0	0	0	0	0	0	1	0	0
M	46	0	0	0	0	0	0	0	0	0	0	0	0	0	0	***43***	0	0	0	0	0	0	2	0	0	0	0	0	0	0	0	1	0
O	15	0	0	0	0	0	0	0	0	0	0	0	0	0	0	0	***15***	0	0	0	0	0	0	0	0	0	0	0	0	0	0	0	0
Sum	909	22	14	72	10	20	72	10	36	76	199	134	81	2	15	63	15	34	5	4	3	2	2	2	2	2	2	1	1	1	1	1	5

* The corresponding genomovars of italic numbers were focus-specific Major genomovars

From Table 1, we can also see the region-specific distribution of genomovars in different foci. For instance, genomovar13 strains were only found in Focus J, and genomovar15 ones exclusively in Focus O, while genomovar09 ones mainly in Focus F. On the other hand, strains in a specific focus always fell into a few genomovars. For example, all the 15 strains from Focus O belonged to genomovar15. While 191 of 198 strains from Focus F were identified as genomovar09, and the other 7 strains fell into other 5 genomovars, with no more than 3 strains each. The numbers of strains, belonging to different genomovars that predominated in certain plague foci, were italicized in Table 1.

Based on the data in Table 1, we coined the terms, Major genomovar and Minor genomovar, to describe the regional specificity of genome plasticity for *Y. pestis* strains. When looking into the background of these strains, we found that almost all the strains belonging to the Major genomovars in a specific focus were isolated from the main hosts and vectors and distributed throughout the focus, whereas the Minor genomovar strains were isolated mainly from the minor hosts and distributed sporadically along the border of neighboring foci. Based on the natural foci and adaptive evolution theories, we assumed that Major genomovar strains can well adapt themselves to the ecological environment of the focus and play an important role in conserving the trait of the plague focus. The strains that belong to the Minor genomovars were sporadic in certain foci and might make little contribution to conserve the feature of the focus, but could play roles in a particular stage during adaptive microevolution of *Y. pestis*. They might be eliminated under the pressure of natural selection. However, we still need more evidences to support this hypothesis, especially the phenotypic effects of DFR loss-and-gain during microevolution of *Y. pestis*.

**Figure 2:**
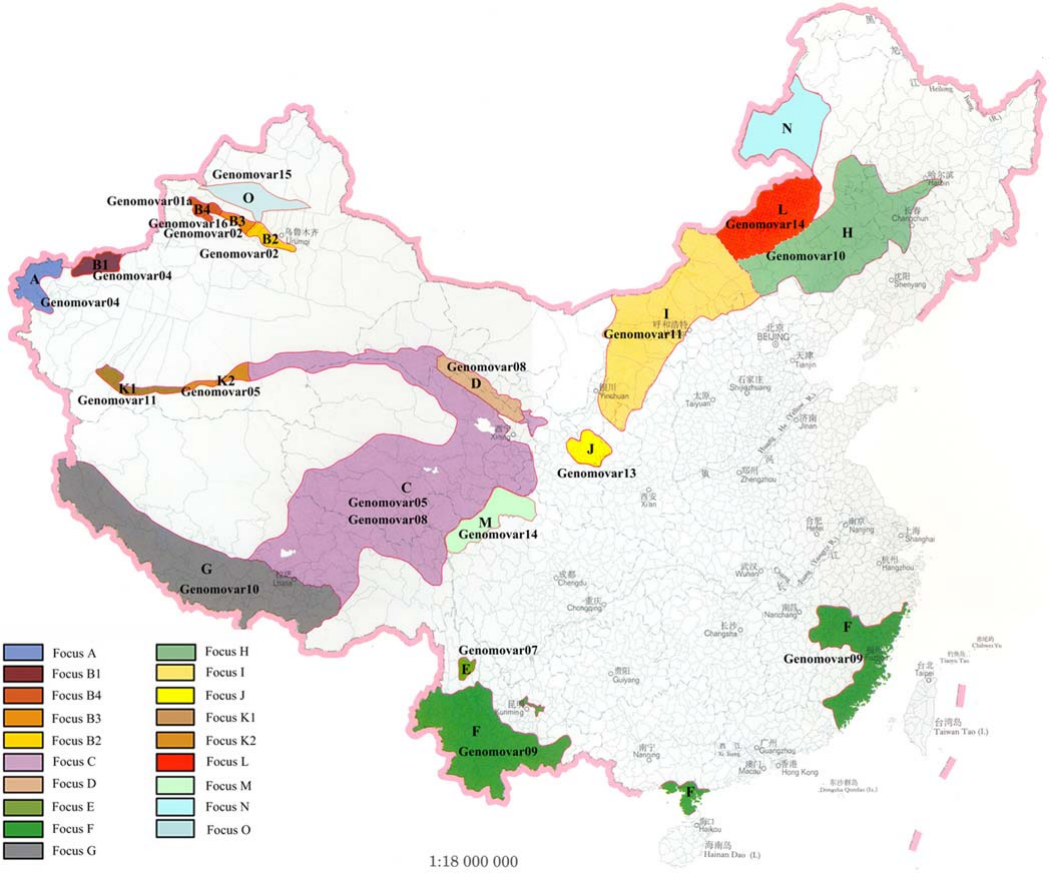
The distribution Major genomovars in natural plague foci of China. There are 15 plague foci in China. Focus A: *Marmota caudate* Plague Focus of the Pamirs Plateau; Focus B: *Marmota baibacina-Spermophilus undulates* Plague Focus of the Tianshan Mountains; Focus C: *Marmota himalayana* Plague Focus of the Qinghai-Gansu-Tibet Grassland; Focus D: *Marmota himalayana* Plague Focus of the Qilian Mountain; Focus E: *Apodemus chevrieri-Eothenomys miletus* Plague Focus of the highland of Northwestern Yunnan Province; Focus F: *Rattus flavipectus* Plague Focus of the Yunnan-Guangdong-Fujian provinces; Focus G: *Marmota himalayana* Plague Focus of the Gangdisi Mountains; Focus H: *Spermophilus dauricus* Plague Focus of the Song-Liao Plain; Focus I: *Meriones unguiculatus* Plague Focus of the Inner Mogolian Plateau; Focus J: *Spermophilus dauricus alaschanicus* Plague Focus of the Loess Plateau in Gansu and Ningxia provinces; Focus K: *Marmota himalayana* Plague Focus of the Kunlun Mountains; Focus L: *Microtus brandti* Plague Focus of the Xilin Gol Grassland; Focus M: *Microtus fuscus* Plague Focus of the Qinghai-Tibet Plateau; Focus N: *Marmota sibirica* Plague Focus of the Hulun Buir Plateau of Inner Mongonia. Focus O: *Rhombomys opimus* Plague Focus of the Junggar Basin of Xinjiang. B1, B2, B3 and B4 are subfoci of Focus B, K1 and K2 are subfoci of Focus K. Focus N is a silent plague focus without strains available for this study.

Notably, Major and Minor genomovars make sense only by combining with the concept of natural plague foci. The Major genomovar in one plague focus might be the Minor one in the other. For example, genomovar09 was the Major genomovar in Focus F, but the Minor one in Focus E. The distribution of the Major genomovar(s) in each plague focus of China was presented in Figure 2. Normally, each focus has its own characteristic Major genomovar(s). However, there were still some strains from several foci indistinguishable by the DFR profiles. For instance, strains from Foci G and H shared genomovar10, Foci K2 and I genomovar11 and Foci L and M genomovar14. This suggested the close relationship between the strains in the corresponding foci. These strains might be recently spread from one focus to another and there was no enough time for DFR varieties to accumulate. We might need other methods with higher resolution to differentiate strains from these foci. Actually, based on CRISPR(clustered regularly interspaced short palindromic repeat) and MLVA(multiple-locus VNTR analysis) analysis we are able to differentiate strains from Foci L and M, as well as K2 and I.

Anyway, with this updated DFR typing system, we can roughly differentiate strains from most plague foci. In another word, we can tell the possible origin of certain *Y. pestis* strain by investigating their DFR profiles using a set of PCRs. We can estimate from the above data that this DFR-based genotyping system should be scientific sound because it correlates very well with the focus distribution of the pathogen and the conventional ecotyping system that is widely used by Chinese plague scientists for typing *Y. pestis* [23]. We also performed genotyping analysis for one-third of the strains used in this study by MLVA, CRISPR, SNPs and IS-based method, validating the DFR-based method for genotyping plague bacteria (unpublished data). This inexpensive method can be developed as a source tracing protocol when unexpected plague outbreaks or bioterrorism attacks happen.

### *In silico* DFR profiling of the sequenced *Y. pestis* and *Y. pseudotuberculosis* genomes

There are now 16 completed or draft genomes of *Y. pestis* and two completed genomes of *Y. pseudotuberculosis*, providing us valuable data to probe into the relationship between *Y. pestis* strains from China and other regions in the world. We investigated the presence or absence of the 23 DFRs in the 18 genomes by Blast searching, and the results were shown in Table 2.

**Table 2:** The DFR profiles of the sequenced and sequencing Y. pestis and Y.pseudotuberculosis

Strain name	Isolate location	Accession number	Biovar	Genomovar or similar Genomovar	DFR profiles																						
					01	02	03	04	05	06	07	08	09	10	11	12	13	14	15	16	17	18	19	20	21	22	23
Nepal516	Nepal	CP000305	Antiqua	19	-	+	+	+	+	-	-	+	+	+	+	+	-	+	-	-	+	-	+	+	+	+	-
B42003004	China	AAYU00000000	Antiqua	01a	-	+	+	+	+	+	+	+	+	+	+	+	-	+	+	+	+	+	+	+	+	+	+
E1979001	China	AAYV00000000	Antiqua	07	-	-	+	-	+	+	+	+	+	+	+	+	-	+	+	+	+	+	+	+	+	+	-
UG05-0454	Uganda	AAYR00000000	Antiqua	04*	-	-	+	+	+	+	+	+	-	+	+	+	-	+	+	+	+	+	+	+	+	+	-
Antiqua	Congo	CP000308	Antiqua	05	-	-	+	+	+	+	+	+	+	+	+	+	-	+	+	+	+	+	+	+	+	+	-
Angola	Angola	AF167310	Antiqua	03*	-	-	-	-	+	+	+	+	-	-	+	-	-	-	+	+	+	-	+	+	+	+	+
KIM	Kurdistan	AE009952	Medievalis	15	-	+	+	-	+	-	-	+	+	-	+	+	-	+	-	-	-	-	+	+	+	+	-
K1973002	China	AAYT00000000	Medievalis	17	-	+	+	+	+	-	-	+	+	+	+	+	-	+	-	-	-	-	+	+	+	+	-
91001	China	AE017042	Microtus	14	+	+	+	+	+	-	+	+	+	+	-	-	-	+	+	+	+	-	-	-	+	+	-
F1991016	China	ABAT00000000	Orientalis	09	-	-	+	-	+	+	+	+	+	+	+	+	+	+	+	+	+	+	+	+	+	+	-
MG05-1020	Madagascan	AAYS00000000	Orientalis	09*	-	-	+	-	+	+	+	+	+	-	+	+	+	+	+	+	+	+	+	+	+	+	-
FV-1	Arizona.USA	AAUB00000000	Orientalis	09*	-	-	+	-	+	+	+	+	+	+	+	+	+	+	+	-	+	+	+	+	+	+	-
CA88-4125	California.USA	ABCD00000000	Orientalis	09	-	-	+	-	+	+	+	+	+	+	+	+	+	+	+	+	+	+	+	+	+	+	-
CO92	Colorado.USA	AL590842	Orientalis	09	-	-	+	-	+	+	+	+	+	+	+	+	+	+	+	+	+	+	+	+	+	+	-
IP275	Madagascan	AAOS00000000	Orientalis	09*	-	-	-	-	+	-	-	-	+	-	+	+	-	+	+	+	+	+	+	+	-	+	-
Pestoides F	Former Soviet Union	AF167309	Pestoides	30*	+	+	+	-	+	-	-	+	-	+	+	+	-	+	+	+	+	-	+	+	+	+	+
IP 32953	France	BX936398	*Y. pseudotuberculosis*	14*	-	-	-	+	+	-	+	+	+	+	+	-	-	+	+	+	+	-	+	+	+	+	-
IP 31758	Former Soviet Union	CP000720	*Y. pseudotuberculosis*	14*	-	-	-	+	+	-	+	+	+	+	+	-	-	+	+	+	-	-	-	+	+	+	-

* : The most similar genomovars to the corresponding strains

+: The presence of the DFR

-: The absence of the DFR

Ten of the 16 *Y. pestis* genomes, 91001, Nepal516, B42003004, E1979001, Antiqua, KIM, K1973002, F1991016, CA88-4125 and CO92, fell into the 32 genomovars identified in this study. In these strains, Nepal516, Antiqua[24], KIM[25], CO92[26] and CA88-4125[27] were isolated outside China. The other 6 strains failed to be classified into any of the 32 genomovars, and hence they should be grouped as new genomovars which need to be verified by using more strains. IP275[28], FV-1[29] and MG05-1020 were quite similar to genomovar09 with only one or two DFR differences. UG05-0454 was different from genomovar04 only by the absence of DFR02. Strains Angola and Pestoides F[30] were very different from Chinese strains by DFR profiling. Although the sequenced genomes were obviously not enough to cover the varieties of DFR profiles of *Y. pestis* strains worldwide, we tried to get some interesting results from these limited data.

### Distribution of DFR23 in *Y. pestis*

In our previous study, we identified a 383bp region (DFR4) specific for the *Y. pestis* strains from plague Focus B in China by suppression subtractive hybridization (SSH)[21]. We also confirmed the presence of this fragment in *Y. pseudotuberculosis* strains 53518 (serotype I), 53519 (serotype II) and 29833 (serotype I)[21]. We renamed it as DFR23 in this study in order to maintain the consistency of our DFR nomenclature system.

DFR23 was thought to be specific to *Y. pestis* from Focus B in our previous study by using limited number of strains[21]. In this study, DFR23 were found in all the isolates of Subfoci B2 (46 Antiqua strains), B3 (71 Antiqua strains) and B4 (17Antiqua strains). Whereas 11 of 14 Antiqua strains of Subfoci B1 did not possess this DFR. Meanwhile, 10 strains from Foci A, C, D, K and M harbored this region. Obviously, DFR23 was not only present in the strains from Focus B. The 4 strains from Foci C and D harboring DFR23 were thought to be the most ancient strains of China according to our MLVA results (unpublished data). As DFR23 was also present in some strains of *Y. pseudotuberculosis*, we presumed that the ancient *Y. pestis* strains should possess this region, and it was lost during the microevolution of *Y. pestis*. Interestingly, DFR23 also presented in the sequenced *Y. pestis* strain Angola and Pestoides F. Pestoides F was thought to be a very ancient strain, because it had a *Y. pseudotuberculosis* genomic region that was absent in all known *Y. pestis* strains. It seemed that DFR23 loses in the early phase of microevolutionary history of *Y. pestis*, and it might serve as a marker for identification of ancient *Y. pestis* strains.

### The emergence and microevolution of the Orientalis strains

In this study, 205 Chinese Orientalis strains were grouped into 3 genotypes: genomovar 09, 18 and 25(Figure 1). 199 strains (98%) fell into genomovar09, whereas only 4 and 2 strains the genomovar18 and 25, respectively. These two Minor genomovars (genomovar18 and 25) were therefore not considered in inferring the relationships among Orientalis strains.

The third plague pandemic, caused presumably by the strains of biovar Orientalis, was believed to have originated from Yunnan Province, China, in 1855[31]. It then spread around the world with the aid of modern transportation[32]. Our studies strongly supported this notion. The Orientalis strains in China were mainly isolated from Focus F and grouped into genomovar09. Most Antiqua strains in Focus E, a neighbor to Focus F, fell into genomovar07, and only a few Orientalis strains genomovar09. It suggested that the Orientalis strains of genomovar09 be evolved from genomovar07, the Antiqua strains, in Focus E after acquiring DFR13 and other unknown genetic variations, and then expanded to Focus F even all over the world[20]. The sequenced Orientalis strain CO92[26] and CA88-4125[27], as well as F1991016 isolated from Focus F in China have identical DFR profiles with genomovar09. We hypothesized that Orientalis strains MG05-1020, IP275 and FV-1 came into being by losing certain DFRs from genomovar09.

**Figure 3:**
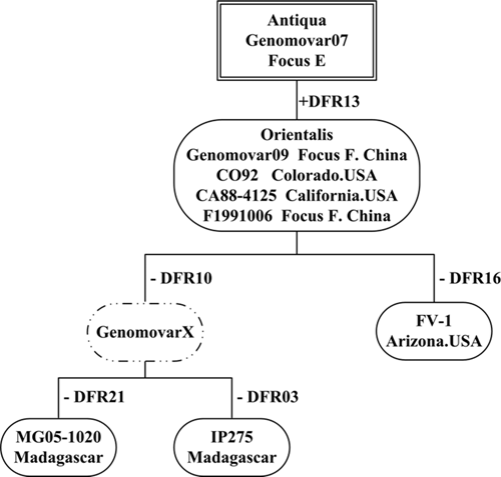
The microevolution scenario of Orientalis strains based on the gain-and-loss of DFRs. Orientalis strains evolved from genomovar07 Antiqua strains of Focus E, by acquiring DFR13, and then evolved as different genomovars by losing certain DFRs. A virtual genomovarX was proposed to illustrate the step-by-step reductionism evolution.

By Comparing DFR profiles of the 6 sequenced Orientalis strains and the tested Orientalis strains in China, we deduced the microevolutionary pattern of Orientalis strains based reductionism (Figure 3). We also proposed a virtual genomovarX as the missing link of genomovar09 to MG05-1020 and IP275. Interestingly, MG05-1020 and IP275 were both isolated in Madagascar. It is hopeful that we can find this genomovar in Madagascar or elsewhere, which will be of great help for better understanding the spreading of the third plague pandemics.

The Orientalis is believed to be “young” biovar of *Y. pestis*, and the time for its spreading all over the world is no longer than 120 years. We can see that Orientalis strains in China were very homologous in DFR pattern (only 3 genomovars in 205 strains with 199 strains as genomovar09) and strains outside China showed considerable heterogeneity (4 genomovars in 5 strains). One possibility was that, when they expanded all around the world, the genome underwent mutations including parallel loss of DFRs for adapting themselves to various niches. The adaptive microevolution might lead to the discrete segregation between the progenitor and offspring strains. This genome reduction gradually caused the offspring strains to inhabit a more specific host niche, without overlapping with their progenitors.

### *Y. pestis* Microtus strains seems to be closely related to *Y. pseudotuberculosis*

In our previous study, we assumed an ancestor *Y. pestis* strain as DFR12 positive[20]. However, we found in this study that, DFR12 was shared by almost all *Y. pestis* strains but genomovar14 (Microtus) and genomovar20 (Medievalis), as well as strain Angola. DFR12 was also absent from genomes of *Y. pseudotuberculosis*. Genomovar20 (only two strains) was a Minor genomovar which might contribute little to the microevolution of the *Y. pestis,* and DFR12 in these two genomes might lose under certain unexpected conditions. Therefore, we neglected genomovar20 in discussing the DFR12 issue. If the ancestor *Y. pestis* strain was DFR12 positive, genomovar14 and strain Angola must abandon DFR12 from their genomes again, which can not be well explained by maximum parsimony principle in the evolution. So it might be more convincing to set a virtual ancestor *Y. pestis* strain as DFR12 negative.

Most Microtus strains in China were isolated from Foci L and M. In this study, 95.5% of Microtus strains as well as 91001 fell into genomovar14, which was Major genomovar of Foci L and M. From Table 2, we can see that, genomovar14 was quite similar to *Y. pseudotuberculosis* IP32953 and IP31758 (by only differing in 3 DFRs except for the DFRs located in plasmid pMT1). We deduced that, from the point view of DFR profiling, strains belong to genomovar14 (including 91001) might be those most closely related to *Y*. *pseudotuberculosis*, which has been proved by SNPs analysis[3].

### Diversity of *Y. pestis* strains in Xinjiang province

Xinjiang is the biggest province of China. Its complex terrains and landforms as well as a wide variety of ecological systems have created a natural paradise for biodiversity. The *Y. pestis* in this region presented highly diversity in their genomes according to the DFR profiles. Of the 32 genomovars, 13 were identified in this province and 7 of them were Major genomovars (Table 3). The significant diversity implied a long evolutionary history of *Y. pestis* in this region, or the *Y. pestis* in China might be originated from this region.

**Table 3:** The distribution of genomovars of *Y. pestis* in the foci of Xinjiang

Focus or Subfocus	Number of strains	Genomovar*												
		01a	01b	02	03	04	05	11	15	16	17	25	28	31
A	13	0	0	0	0	***11***	0	0	0	1	0	1	0	0
B1	14	0	1	3	0	***9***	0	0	0	0	0	0	0	1
B2	46	2	0	***43***	0	0	0	0	0	1	0	0	0	0
B3	71	3	0	***24***	10	0	0	0	0	***32***	0	0	0	2
B4	17	***15***	0	2	0	0	0	0	0	0	0	0	0	0
K1	14	1	0	0	0	0	0	***12***	0	0	0	0	1	0
K2	11	1	1	0	0	0	***6***	0	0	0	1	0	0	2
O	15	0	0	0	0	0	0	0	***15***	0	0	0	0	0

* : The corresponding genomovars of the italic numbers are dominant in a certain focus, and determined as Major genomovars in certain focus.

There are 4 foci found in Xinjiang to date, including Foci A, B (Subfoci B1 to B4), O and K (Subfoci K1 and K2). The first 3 are geographically linked to the plague foci of the Central Asia (See Figure S1 ) [7]. Due to the lack of the strains outside China, it is still very difficult to provide a detailed and integrated relationships between the strains in Xinjiang and those of the Central Asia. The Figure S1 showed that Plague Foci in the Desert of Central Asia (labeled with “1” ) stretched eastward directly to China, and jointed with the newly identified Focus O, *Rhombomys opimus* (great gerbil) Plague Focus of the Junggar Basin of Xinjiang [33]. The Foci B (Subfoci B1–B4) and A in China adjoin Plague Foci of Western Section of Tianshan Mountains (labeled with “3”) and Plague Foci of Pamirs-Alai (labeled with “2”), respectively. The close geographical relationships implied that, Xinjiang province might act as an exchange access of *Y. pestis* between China and Central Asia. In 2005, 15 strains were isolated from the *Rhombomys opimus* in Focus O[33]. All of them were biovar Medievalis and grouped into genomovar15, the same as the sequenced Medievalis strain KIM isolated from Kurdistan[25]. It is very unusual for strains apart so far away to share the same DFR profile. It is still difficult to tell the direction of foci expansion, and we need strains from former USSR to figure out the exact evolution scenario of *Y. pestis* in this region.

By comparing the DFR profiles of Xinjiang strains, the microevolution of the genomovars seemed to be consistent with the expansion of plague foci. Subfocus B4 situates in the west section of the Northern Tianshan Mountain (NTM) and joins with the plague foci of Kazakstan, where the Major genomovar is genomovar01a (similar to the hypothetical ancestor of *Y. pestis*). The strains isolated from Subfocus B3, the mid-section of the NTM, fell into genomovar 02 and 16, and they two were equally defined as the Major genomovars. The east section of the NTM was designated as Subfocus B2 where the Major genomovar is genomovar02. There is no geographical obstacle between these 3 subfoci, which might account for the spreading of *Y. pestis* from west to east. By reductive evolution hypothesis, we proposed that when the strains of genomovar01a transmited from B4 to B3, DFR10 was lost in their genomes to adapt the new niches and colonized as genomovar02, then expanded to B2 and stably existed as the Major genomovar there. Genomovar16 came into being after losing DFR04 and played an important role together with genomovar02 within Subfocus B3. Then different genomovars evolved continuously by losing or acquiring certain DFRs to adapt to new niches of the host.

The great genomic diversities of Xinjiang strains make them an ideal collection to study the microevolution of *Y. pestis*, some other markers such as SNP, VNTR and CRISPR might help us better understand the myth of its evolution.

### Distribution of DFR 13 in 4 *Y. pestis* biovars

DFR13 potentially encodes a prophage (Ypf*Φ*), which contains 13 putative open reading frames (ORFs) (YPO2271–2281)[34]. It was previously suggested that DFR13 was restricted to the Orientalis strains[19], [20], [35]. However, recent studies indicated that it was acquired by the *Y. pestis* ancestor, and its genome presented in the three *Y. pestis* biovars[34]. Our results in this study strongly support this notion. Of the 377 strains amplified with 3 primer pairs targeting different regions of DFR13, all 52 Orientalis strains were positive for all 3 loci. Meanwhile, 6 out of 222 Antiqua strains, 1 out of 60 Medievalis and 1 out of 43 Microtus strains were also positive for at least 2 loci, although the amplicons were somehow faint (Table 4). The sequences of the positive products were highly homologous to the corresponding region of CO92 by sequencing analysis (data not shown). It was supposed that the phage may not be stably integrated in the genomes of non-Orientalis strains and the proportion of phage-positive cells within the bacterial population may be unequal[34]. The serial dilution was used for confirming the content of this phage DNA in *Y. pestis*. If its content is lower than the chromosomal DNA it should become negative by PCR after a certain dilutions. After diluting the DNA template 8 times, we failed to identify any amplicon from these 8 non-Orientalis strains. This suggested that the signal variations detected among various *Y. pestis* strains may be due to a difference in the proportion of phage-positive cells within the bacterial population. It also implied that, in contrast to Orientalis strains, the phage is unstable in the other three biovars and easy to lose under laboratory conditions. One Microtus strain M1997002 was identified to harbor the phage in this study, which strongly supported the suggestion that the Ypf*Φ* had been acquired horizontally, as an unstable episome by the *Y. pestis* ancestor after its divergence from *Y. pseudotuberculosis*. The phage then became stable in the Orientalis strains, upon permanent integration of its genome into the bacterial chromosome[34].

**Table 4:** Non-Orientalis strains amplified with DFR13 identification primers

Strain	Biovar	Isolate location	PCR(2ng/µl)*			PCR(0.25ng/µl)*		
			YPO2274	YPO2277	YPO2273	YPO2274	YPO2277	YPO2273
D0000002	Antiqua	Qinghai qilian	+	+	-	-	-	-
K21985006	Antiqua	Xinjiang ruoqiang	+	+	+	-	-	-
A1956001	Antiqua	Xinjiang wuqia	+	+	+	-	-	-
B11979001	Antiqua	Xinjiang atushi	+	+	-	-	-	-
B31989002	Antiqua	Xinjiang wusu	+	+	-	-	-	-
M1997002	Microtus	Sichuan shiqu	+	+	-	-	-	-
I1978002	Medievalis	Inner Mongolia guoqianqi	+	+	-	-	-	-
H1955008	Antiqua	Inner Mongolia keyouqianqi	+	+	+	-	-	-
EV76	Orientalis	Its parent strain was isolated from a patient in Madagascar	+	+	+	+	+	+

* : Indicated the template concentration

The “+” and “-” indicated the positive and negative results, respectively.

EV76 was the positive control.

### Concluding remarks

In this study, 909 strains of *Y. pestis* from China were grouped into 32 genomovars. Orientalis, Medieavalis and Microtus strains showed biovar specific DFR profiles, and were clustered into three distinct groups. But genomovars of Antiqua strains distributed among these three groups. Genomovars distribution was somehow focus-specific in China, and we proposed Major and Minor genomovars for explaining their distribution and roles played in microevolution of *Y. pestis*. By *in silico* DFR profiling of the sequenced genomes, we were able to compare Chinese strains and those outside China as well. Orientalis strains in China turned out to be more ancient than those aboard according to the DFR profiles, supporting the notion that the Orientalis strains were originated from China. Xinjiang province could be an access of *Y. pestis* spreading between China and Central Asia. It is the first time that we systematically classified a large amount of strains in China based on the profiling gene acquisition/loss in their genomes. Data presented here will be of great help to develop a genomic polymorphism database of *Y. pestis* for tracing the origin of this agent when the plague outbreak or bioterrorism attack occurs. Hopefully, DFR analysis can be modified for genotyping other bacteria that have similarly plastic genomes.

## Materials and Methods

### Strains and DNA

912 strains isolated from 15 plague foci from the year of 1943 to 2005 were included in this study, which presumably represented the most abundant diversity of *Y. pestis* strains in China. All the strains were collected by Qinghai Institute for Endemic Diseases Prevention and Control, the Center for Disease Control and Prevention of Xinjiang Uygur Autonomous Region and the Yunnan Institute for Endemic Disease Control and Prevention. The bacteria were cultivated in nutrient agar at 28°C for 48 hours, and then the genome DNAs were extracted by using conventional SDS lysis and phenol-chloroform extraction method.

### Genotyping based on DFR profiling

As three (DFR 01-03) of the 23 DFRs are located on plasmid pMT1, all the strains were screened by pMT1-specific primers before genotyping. Primers AP-YPMT1.44F (5′AACACTATCTCATTCCGCAGTAAAG3′) and AP-YPMT1.44R (5′AGTGGATGATGAAGTAGACCGAG3′) were used to screen the presence or absence of this plasmid. The primers used for amplify the 23 DFRs were provided in the Table S1. The composition of PCR mix and the reaction conditions were describe elsewhere[20], [21]. The DNA mixture of strains 91001 and EV76 were used as positive control. Negative control was also set in each plate to monitor the amplification.

The data were processed with Bionumerics 5.00 (Applied Math NV. Belgium). Dendrogram was constructed by the Neighbor-Joining method with Dice means.

### *In silico* DFR typing of the published genomes

Sequence blasting was performed on NCBI to compare the PCR target genes of the 23 DFRs with the genomes of the sequenced and sequencing *Y. pestis* and *Y. pseudotuberculosis*. The gene was thought to be present if the identities between the query and subject sequences were above 98%, with 95% coverage of the gene.

### Distribution of DFR13 in 4 *Y. pestis* biovars

To evaluate the distribution of DFR13 in the 4 biovars strains, 377 strains (60 Medievalis, 43 Microtus, 222 Antiqua and 52 Orientalis) were tested with the primers for DFR13 amplification. Primers targeting YPO2273, YPO2274 and YPO2277 were used[34].

For the positive amplicons obtained from biovars other than Orientalis, they were sequenced and compared with the corresponding regions of CO92. Furthermore, a two-fold serial dilution (starting from 2 ng/µl) of DNA from the positive strains and strain EV76 were prepared and used as templates for amplification as mentioned above in order to evaluate the relative contents of the specific target.

## Supporting Information

Table S1 Primers used for DFR analysis
(0.09 MB DOC)

Figure S1 The geographic relationship between the foci in Xinjiang and Central Asia. 1. Plague Foci in Desert of Central Asia. 2. Plague Foci of Pamirs-Alai. 3. Plague Foci of Western Section of Tianshan Mountains. Foci A, B1–B4 and O see Figure 2
(3.17 MB TIF)
