# Supplementary material for: Different Region Analysis for Genotyping Yersinia pestis Isolates from China
Source: PLoS One. 2008 May 14;3(5):e2166. doi: 10.1371/journal.pone.0002166 (PMC2367435; doi:10.1371/journal.pone.0002166)
Supplement: Table S1 — Primers used for DFR analysis (0.09 MB DOC) [file pone.0002166.s001.doc]

# Primers used for DFR analysis

| DFR | Primer name | Primer sequences | Position | Amplicon (bp) |
| --- | --- | --- | --- | --- |
| DFR01 | AP-pMT046F | 5’AGAGAGTTTCATCTATCAGACCATG 3’ | 813-838 | 200 |
|  | AP-pMT046R | 5’ATACCAGTGGATCGTCTTTGATTTC 3’ | 1012-987 |  |
| DFR02 | AP-pMT090F | 5’CATCCGTTCTACATCATCCATAGC 3’ | 883-907 | 300 |
|  | AP-pMT090R | 5’CCAGATCTCATCCAGGTACTTATG 3’ | 1182-1158 |  |
| DFR03 | AP-YPMT1.06cF | 5’AGCACAAGCAGATGGTCAATAATG 3’ | 3107-3131 | 400 |
|  | AP-YPMT1.06cR | 5’TGTGCCTTCAGTTGGGTAATTTG 3’ | 3506-3483 |  |
| DFR04 | AP-YP0976F | 5’GTGGAGTACCTCTTATCTGGATG 3’ | 582-605 | 500 |
|  | AP-YP0976R | 5’CAAATATTTCACCGCGTTTAACC 3’ | 1081-1058 |  |
| DFR05 | AP-YPO0624F | 5’TAATATACCTCTCGCAGAAAGCAG 3’ | 678-702 | 400 |
|  | AP-YPO0624R | 5’GCCAATAGCAATACACCATTCTG 3’ | 1077-1054 |  |
| DFR06 | AP-YPO0739F | 5’GAAAATCATCGAGCGCTACTGG 3’ | 566-587 | 318 |
|  | AP-YPO0739R | 5’GGAATGTGGCTTCTGCCTTG 3’ | 883-864 |  |
| DFR07 | AP-YPO0743F | 5’TGTGTCACCAATGGCACTTAAAC 3’ | 324-347 | 200 |
|  | AP-YPO0743R | 5’GGCTATCTATCTGCACCTGACTC 3’ | 523-500 |  |
| DFR08 | AP-YPO0988F | 5’ACCGTTTACGCCTCAATATGTTG 3’ | 647-670 | 300 |
|  | AP-YPO0988R | 5’GATAGAATAATACCAGCGGTTGAAC 3’ | 946-921 |  |
| DFR09 | AP-YPO1002F | 5’TATGTGCCGTCATCTATCAAGTC 3’ | 168-191 | 400 |
|  | AP-YPO1002R | 5’TTTAACTAAATCATCCACCTCACAG 3’ | 567-542 |  |
| DFR10 | AP-YPO1168F | 5’GTTACCGTTCAGTTTTGTGATTTTC 3’ | 1425-1450 | 500 |
|  | AP-YPO1168R | 5’TCTGTTCTTTGCTGTAGTCCATC 3’ | 1924-1901 |  |
| DFR11 | AP-YPO1987F | 5’CTGGAAAATGCCCTACCG 3’ | 71-88 | 1553 |
|  | AP-YPO1987R | 5’TCGGTCGGCTTTATCCC 3’ | 1623-1608 |  |
| DFR12 | AP-YPO2110F | 5’TTGAAGTTGATGGCAAGAAAACC 3’ | 455-478 | 300 |
|  | AP-YPO2110R | 5’CATTGATTGGATATGAGCGGAAG 3’ | 754-731 |  |
| DFR13 | AP-YPO2271F | 5’GTTCTCCAGTTGTAGGTG 3’ | 560-577 | 191 |
|  | AP-YPO2271R | 5’ATTCGTCACAGTGCGTTC 3’ | 750-733 |  |
| DFR14 | AP-YPO2286F | 5’CCGATCTTAATCAGGCTCTTCAG 3’ | 545-568 | 300 |
|  | AP-YPO2286F | 5’CTTGCGAGGTAATTTGGTTCTTG 3’ | 844-821 |  |
| DFR15 | AP-YPO2315F | 5’GATTTTGATGGTTCTTTCACATTTG 3’ | 163-188 | 400 |
|  | AP-YPO2315R | 5’CTTCTACTGACAGGATCAATTCG 3’ | 562-539 |  |
| DFR16 | AP-YPO2375F | 5’CTCATCTGCATACCGACTATCTG 3’ | 344-367 | 500 |
|  | AP-YPO2375R | 5’GGTCATGGTCAGAGAAAGTGATG 3’ | 843-820 |  |
| DFR17 | AP-YPO2380F | 5’ATGGCAATGTTATCAGCATGGAG 3’ | 1799-1822 | 400 |
|  | AP-YPO2380R | 5’GTATAAATACCCGCTTCCCTTACG 3’ | 2198-2174 |  |
| DFR18 | AP-YPO2469F | 5’AAGTGGAATGGCTATTGC 3’ | 74-90 | 267 |
|  | AP-YPO2469R | 5’GACTGGCGAACAATGTC 3’ | 340-324 |  |
| DFR19 | AP-YPO2489F | 5’GTTTGATATTAAGTGGGC 3’ | 51-68 | 422 |
|  | AP-YPO2489R | 5’AATAAGTTTGCCAGTTTC 3’ | 472-457 |  |
| DFR20 | AP-YPO3047F | 5’GAGACATTCCTGCCTGAGTTATTG3’ | 490-514 | 300 |
|  | AP-YPO3047R | 5’GATAGCCTCATCGGTAAGTTGATC3’ | 789-765 |  |
| DFR21 | AP-YPO3674F | 5’ACGATGCACTGTATCAGCTTATC3’ | 1259-1282 | 400 |
|  | AP-YPO3674R | 5’AGATAACTTTCGCTGTCACTGATG3’ | 1658-1634 |  |
| DFR22 | AP-YPO4017F | 5’TGATGAATAACCTCGATCCTGACG3’ | 101-125 | 500 |
|  | AP-YPO4017R | 5’TTGTTGGCATTCGATGTTCAGAG3’ | 600-577 |  |
| DFR23 | DFR383F | 5’-GTTACAGGAACCTCAGCG-3’ | 20-37 | 364 |
|  | Antiqua-R | 5’-TCCGCAGCAGCAAATTCAC-3’ | 383-365 |  |
