# Supplementary figures and images for: Different Region Analysis for Genotyping Yersinia pestis Isolates from China
Source: PLoS One. 2008 May 14;3(5):e2166. doi: 10.1371/journal.pone.0002166 (PMC2367435; doi:10.1371/journal.pone.0002166)

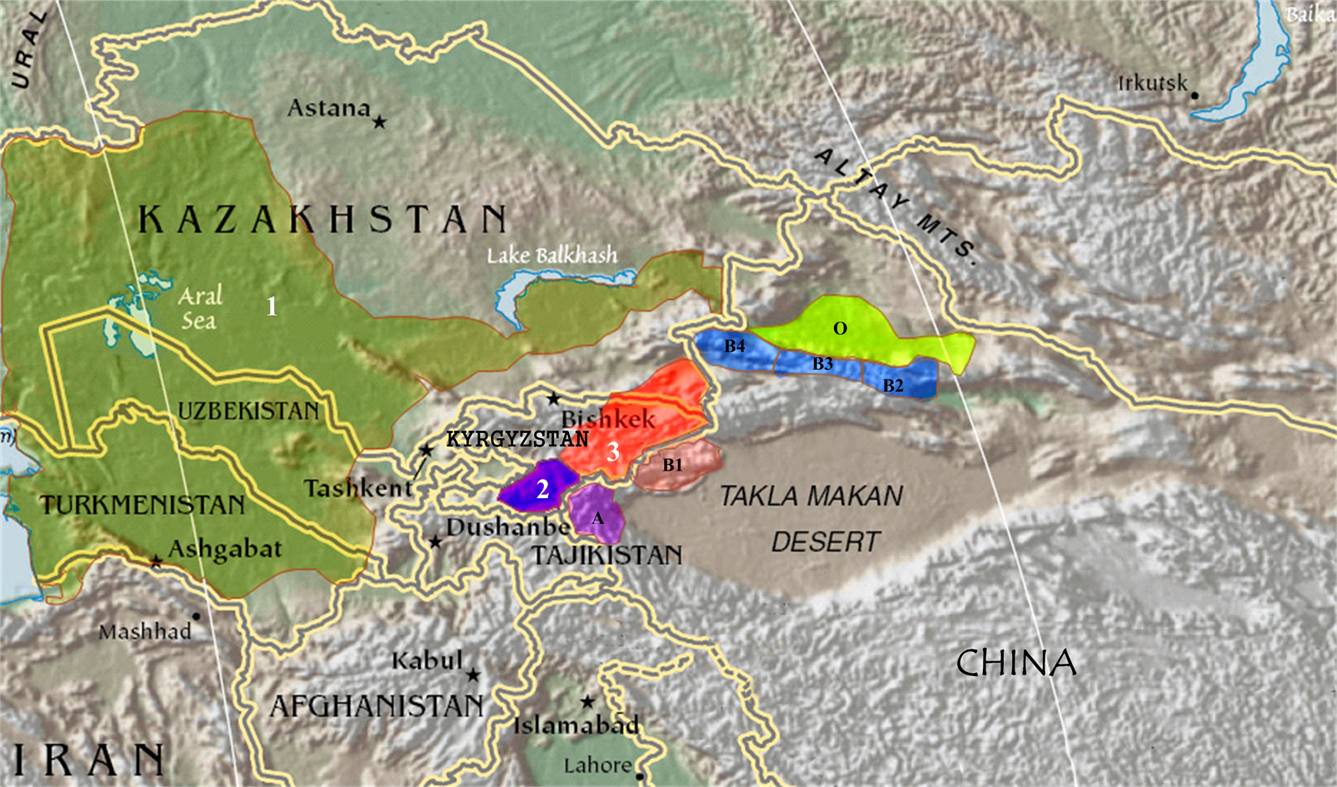

Supplement: Figure S1 — The geographic relationship between the foci in Xinjiang and Central Asia. 1. Plague Foci in Desert of Central Asia. 2. Plague Foci of Pamirs-Alai. 3. Plague Foci of Western Section of Tianshan Mountains. Foci A, B1–B4 and O see Figure 2 (3.17 MB TIF) [file pone.0002166.s002.tif]
